# Indoleacetate decarboxylase is a glycyl radical enzyme catalysing the formation of malodorant skatole

**DOI:** 10.1038/s41467-018-06627-x

**Published:** 2018-10-11

**Authors:** Dazhi Liu, Yifeng Wei, Xuyang Liu, Yan Zhou, Li Jiang, Jinyu Yin, Feifei Wang, Yiling Hu, Ankanahalli N. Nanjaraj Urs, Yanhong Liu, Ee Lui Ang, Suwen Zhao, Huimin Zhao, Yan Zhang

**Affiliations:** 1 Tianjin Key Laboratory for Modern Drug Delivery & High-Efficiency, Collaborative Innovation Center of Chemical Science and Engineering, School of Pharmaceutical Science and Technology, Tianjin University, 300072 Tianjin, China; 2 Metabolic Engineering Research Laboratory, Institute of Chemical and Engineering Sciences, Agency for Science, Technology and Research (A*STAR), Singapore, 138669 Singapore; 3 iHuman Institute, ShanghaiTech University, 201210 Shanghai, China; 4 School of Life Science and Technology, ShanghaiTech University, 201202 Shanghai, China; 5 Technical Institute of Physics and Chemistry, Chinese Academy of Sciences, 100190 Beijing, China; 6 Department of Chemical and Biomolecular Engineering, University of Illinois at Urbana-Champaign, 600 South Mathews Avenue, Urbana, IL 61801 USA

## Abstract

Skatole is a malodorous compound that contributes to the characteristic smell of animal faeces. Although skatole has long been known to originate from bacterial tryptophan fermentation, the enzyme catalysing its formation has so far remained elusive. Here we report the use of comparative genomics for the discovery of indoleacetate decarboxylase, an O_2_-sensitive glycyl radical enzyme catalysing the decarboxylation of indoleacetate to form skatole as the terminal step of tryptophan fermentation in certain anaerobic bacteria. We describe its biochemical characterization and compare it to other glycyl radical decarboxylases. Indoleacetate decarboxylase may serve as a genetic marker for the identification of skatole-producing environmental and human-associated bacteria, with impacts on human health and the livestock industry.

## Abstract (web summary)

Skatole is a bacterial metabolite responsible for boar taint and the objectionable smell of manure. Here, the authors elucidate the final step of skatole biosynthesis, describing the discovery and biochemical characterization of the enzyme catalysing the conversion of indoleacetate into skatole.

## Introduction

**Fig. 1:**
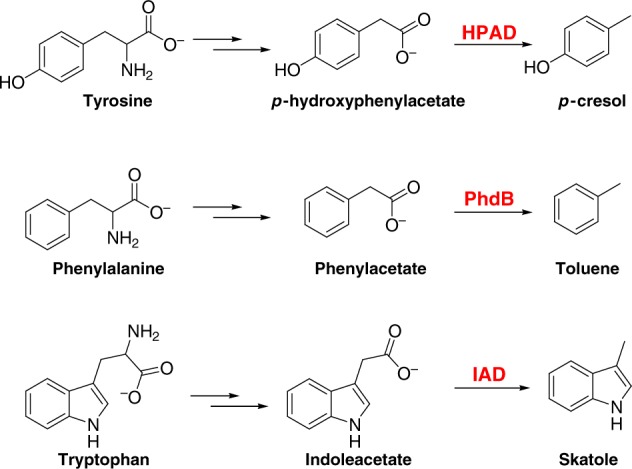
Pathways for fermentation of aromatic amino acids. Tyrosine (Tyr), phenylalanine (Phe), and tryptophan (Trp) are converted into cresol, toluene, and skatole, respectively. HPAD *p*-hydroxyphenylacetate decarboxylase, PhdB phenylacetate decarboxylase, and IAD indoleacetate decarboxylase

Fermentation of aromatic amino acids by anaerobic bacteria leads to a large variety of products that retain their stable aromatic rings (Fig. 1)^1,2^. When produced by bacteria living in the anaerobic human/animal gut, these compounds can accumulate in the host bloodstream, reaching sub-millimolar concentrations and have global physiological or pathological effects^1,3,4^. Therefore, a detailed understanding of these fermentation pathways and their products is vital for human health. Many fermenting bacteria are able to degrade the aromatic amino acids tyrosine (Tyr), phenylalanine (Phe), and tryptophan (Trp) to form *p*-hydroxyphenylacetate, phenylacetate, and indoleacetate, respectively. Some bacteria are able to carry out an additional step, involving the chemically challenging decarboxylation of these compounds to form the volatile aromatic compounds cresol^5^, toluene^6,7^, and skatole^8^.

Of these three volatile products, skatole is the most noticeable, having a distinct faecal malodour detectable at a threshold of 0.00056 ppm (0.0030 mg/m^3^) (cresol, which also has an objectionable odour, is detectable at a threshold of 0.00186 ppm (0.0082 mg/m^3^))^9^. Skatole has long been known to originate from bacterial metabolism^8^, and the biochemical pathway for its production is of considerable interest to the farming industry as skatole is a major component of the objectionable smell of manure, and contributes to boar taint^10,11^ and bovine respiratory diseases^3,12^. Skatole of bacterial origin is also found in human faeces and in humans, it was also found to be a pneumotoxin^13,14^, a possible pulmonary carcinogen^15^, and a partial aryl hydrocarbon receptor agonist^16^. Furthermore, as an oviposition attractant for *Culex* mosquitoes, skatole contributes to the propagation and outbreak of insect-borne human infections such as filariasis, Japanese encephalitis, and West Nile virus^17,18^. However, while the enzymes catalysing cresol^19^ and toluene^6^ formation have been identified, the enzyme catalysing skatole formation has not yet been reported.

The cresol-forming enzyme, *p*-hydroxyphenylacetate decarboxylase (HPAD), was reported in 2001 by Selmer and Andrei^7^, and is a member of the glycyl radical enzyme (GRE) superfamily. This superfamily of enzymes catalyses diverse radical-mediated reactions and plays prominent roles in the primary metabolism of anaerobic-fermenting bacteria^20,21^. Their catalytic mechanism requires an O_2_-sensitive glycyl radical (G•) cofactor, which is generated by an activating enzyme through chemistry involving *S*-adenosylmethionine (SAM) and a [4Fe-4S]^1+^ cluster^22^. Oxygen-sensitive indoleacetate decarboxylase (IAD) activity was previously reported in cell-free extracts of *Clostridium scatologenes*^7^ and a *Lactobacillus* strain^23^, and has been proposed but not demonstrated to be a GRE^7^.

The catalytic mechanism of HPAD has been studied both experimentally and computationally^24,25^, and involves activation of *p*-hydroxyphenylacetate by concerted abstraction of an electron and the phenolic proton to generate a phenoxy-acetate radical anion, with the radical delocalized over the aromatic ring^25^. Because of the different reactivities of the indole and phenyl groups, it is unclear whether the decarboxylation of indoleacetate and phenylacetate could also be catalysed by GREs through analogous mechanisms. Nevertheless, the large number of functionally uncharacterized sequences in the GRE superfamily^20^ (14,288 sequences in the InterPro family IPR004184 to date) prompted us to search for candidate IADs through bioinformatics. While our work was in progress, the toluene-forming enzyme, phenylacetate decarboxylase (PhdB), was reported by Beller et al.^6^ to be a novel GRE, though its catalytic mechanism is unknown at present and likely to differ substantially from HPAD.

The model organism for skatole (and cresol) production is *Clostridium scatologenes* (*Cs*), order Clostridiales, phylum Firmicutes, isolated from acidic sediment^8^. Lately, skatole (and cresol) production was also reported in *Olsenella scatoligenes* (*Os*), order Coriobacteriales, phylum Actinobacteria, isolated from swine manure^26^. The genome sequences of these evolutionarily divergent skatole producers presented the prospect of identifying IAD through comparative genomics, guided by our increasing understanding of the catalytic mechanisms of GREs and key active-site residues involved in the chemistry.

In this work, we report the identification of an IAD in *O. scatoligenes* and its validation through in vitro biochemical assays, adding to the growing chemical repertoire of the GRE superfamily.

## Results

### Identification of a candidate IAD using comparative genomics

**Fig. 2:**
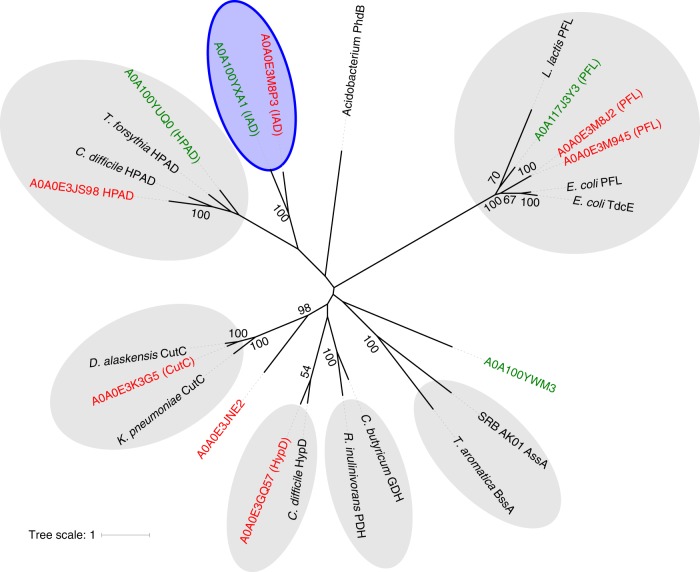
Maximum likelihood phylogenetic tree of GREs. Included are *Cs* GREs (red), *Os* GREs (green), and biochemically validated GREs in other organisms (black). Of the *Cs* and *Os* GREs, only CsHPAD has been previously biochemically validated. Proposed functions of the other *Cs* and *Os* GREs are given in brackets. Candidate IADs are enclosed in the blue ellipse, of which OsIAD was validated in this study. PFL pyruvate formate lyase, TdcE 2-keto acid formate lyase, CutC choline-trimethylamine lyase, PDH propanediol dehydratase, GDH glycerol dehydratase, HypD *trans*-4-hydroxy-l-proline dehydratase, BssA benzylsuccinate synthase, AssA alkylsuccinate synthase, PhdB phenylacetate decarboxylase, HPAD *p*-hydroxyphenylacetate decarboxylase, and IAD indoleacetate decarboxylase reported in this study. Bootstrap confidence values >50 are indicated on the nodes

To identify a candidate GRE with IAD activity, we first sought to annotate the function of all GREs in the genome of *C. scatologenes* (*Cs*) and *O. scatoligenes* (*Os*). *Cs* and *Os* proteins belonging to the InterPro^27^ family IPR004184, which contains the pyruvate formate-lyase domain, were compiled, and a phylogenetic tree of all seven *Cs* and four *Os* GREs, together with selected biochemically validated GRE sequences, was constructed (Fig. 2). The function of several *Cs* and *Os* GREs was inferred by sequence similarity to known GREs and conservation of active-site residues (Fig. 2).

**Fig. 3:**
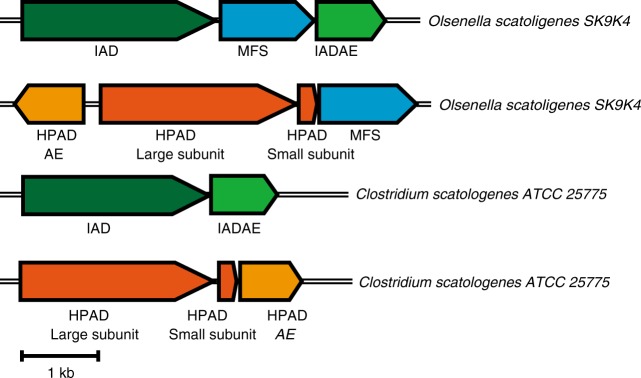
Genome neighbourhood of IAD and HPAD from *Cs* and *Os*. (GenBank accession numbers CP009933 and LOJF01000000 respectively). HPAD *p*-hydroxyphenylacetate decarboxylase, HPADAE HPAD activating enzyme, IAD indoleacetate decarboxylase, IADAE IAD activating enzyme, MFS major facilitator superfamily transporter

We then searched among the remaining unannotated GREs for a candidate IAD common to both *Cs* and *Os*. The proteins A0A0E3M8P3 (*Cs*) and A0A100YXA1 (*Os*) share the greatest sequence identity (51.7%), suggesting that they may share the same function. They also form a branch sister to HPAD, suggesting that they may carry out a mechanistically related decarboxylation reaction. Based on these two observations, these proteins (subsequently referred to as CsIAD and OsIAD) were identified as candidate IADs. Examination of the CsIAD and OsIAD genome neighbourhood (Fig. 3) revealed the presence of putative GRE-activating enzymes. For HPAD, a [4Fe-4S]-containing small subunit was required to form active holoenzyme^19^, and was present in the genome neighbourhood of *Cs* and *Os* HPAD (Fig. 3). In contrast, such a small subunit is absent in the genome neighbourhood of IAD.

### Biochemical assays on the recombinant IAD

To investigate the biochemical activity of the candidate IAD, OsIAD (A0A100YXA1) and its neighbouring activating enzyme OsIADAE (A0A124EH39) were recombinantly produced (Supplementary Fig. 1a, b). OsIADAE was produced with an N-terminal maltose-binding protein (MBP) fusion, as this construct was previously found to increase the soluble expression of a variety of GRE-activating enzymes^20,28,29^. Like most of the other GREs, the purified recombinant OsIAD exists predominantly as a dimer but with a small percentage of monomer (~30%) as analysed by size exclusion chromatography (Supplementary Fig. 1c).

**Fig. 4:**
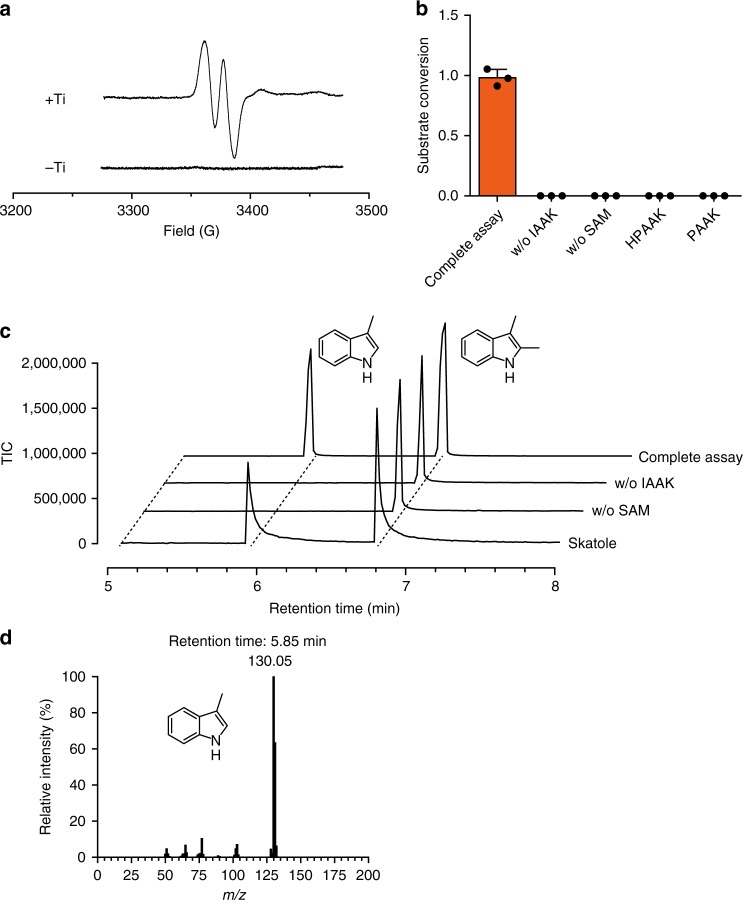
EPR spectra and enzymatic assays of *Os*IAD. **a** X-band EPR spectra of IAD reconstituted with IADAE and SAM in the presence or absence of reductant (Ti(III) citrate). **b** Reaction requirements and substrate specificity of IAD. IAAK, HPAAK, and PAAK are the potassium salts of indoleacetic, *p*-hydroxyphenylacetic, and phenylacetic acids, respectively. (The error bars represent the standard deviation of three individual experiments.) **c** Detection of skatole formation in the IAD-catalysed decarboxylation of IAAK using GC-MS. GC-MS elution profiles of authentic standards of skatole, negative controls omitting SAM and IAAK and a complete assay are displayed as labelled. The internal standard 2,3-dimethylindole is included in each sample. **d** Mass spectrum of the skatole peak

The sequence of OsIADAE contains a conserved CX_2_CX_3_C motif that coordinates the radical SAM [4Fe-4S] cluster^22,30^, as well as a 8-cysteine motif thought to coordinate two auxiliary [4Fe-4S] clusters in a ferredoxin-like domain present in many GRE-activating enzymes (Supplementary Fig. 2)^31^. Anaerobic reconstitution of OsIADAE resulted in 6.5 ± 0.1 Fe and 7.9 ± 0.2 S per monomer (out of a theoretical 12 Fe and 12 S for one radical SAM and two auxiliary [4Fe-4S] clusters) (Supplementary Fig. 3), suggesting a fraction of incompletely reconstituted [3Fe-4S] clusters^32^, and typical UV–Vis spectra for a [4Fe-4S] cluster-containing protein (Supplementary Fig. 4). Like other radical SAM enzymes, OsIADAE cleaved SAM to form 5′-deoxyadenosine in the presence of a strong reductant Ti(III) citrate^19^ (Supplementary Fig. 5). Electron paramagnetic resonance (EPR) spectroscopy showed that OsIADAE could install the G• on OsIAD, forming 0.29 (out of a theoretical maximum of 1)^22^ radicals per dimer (Fig. 4a).

Incubation of activated OsIAD with indoleacetate resulted in the generation of skatole as detected by gas chromatography– mass spectrometry (GC-MS) with reference to an authentic standard (Fig. 4b–d and Supplementary Fig. 6), confirming that OsIAD is indeed an IAD. No activity was detected with phenylacetate or *p*-hydroxyphenylacetate as substrates, indicating high substrate specificity (Fig. 4b). The kinetic parameters of OsIAD were obtained (*k*_cat_  =  2.0 ± 0.1 s^–1^, *K*_M_ = 0.37 ± 0.06 mM) (Supplementary Fig. 7, the error values reported are the standard errors for the fits) and compared to those reported for CsHPAD (*k*_cat_  =  130 s^–1^, *K*_M_ = 0.358 mM)^19^. The two enzymes exhibit a similar *K*_M_, the *k*_cat_ for OsIAD after normalized by radical content, which is ~20-fold slower than that of CsHPAD under optimized reaction conditions.

### Analysis of IAD distribution and genome neighbourhood

To identify IAD homologues from published sequence databases, a sequence similarity network (SSN)^33^ for 14,228 unique sequences in IPR004184 (release 68.0) was constructed using the web-based Enzyme Function Initiative Enzyme Similarity Tool (EFI-EST)^34^, and visualized using Cytoscape v3.5^35^. The *E*-value threshold was adjusted to 10^–260^ (>∼50% sequence identity is required to draw an edge), to place OsIAD and CsIAD within the same cluster (Supplementary Fig. 8). Examination of putative IAD sequences in the IAD cluster (Supplementary Fig. 8) revealed that IAD is present in fermenting bacteria in the orders Clostridiales and Coriobacteriales (Supplementary Fig. 9). IAD is less prevalent than HPAD, and of the 12 unique bacterial species that contain IAD, 8 also contain HPAD. In comparison, PhdB has only been identified in uncultivated bacteria in two metagenomic samples^6^. However, the true prevalence of the three GRE decarboxylases in nature are not necessarily reflected by their prevalence in the sequence databases, which over-represent genomes and metagenomes from cultivatable bacteria and sources related to human health and livestock.

Both the *Os*IAD and HPAD gene clusters include a putative major facilitator family (MFS) transporter (Fig. 3). This MFS is absent in the *Cs*IAD and HPAD gene clusters. Since *Cs* is able to form cresol/skatole from the respective aromatic amino acids^8^, while *Os* is only able to form them from the respective arylacetates^26^, we hypothesize that these MFS transporters are involved in the uptake of the respective arylacetates from the environment. The MFS transporter is also found in the IAD gene clusters of several other organisms, such as *Olsenella uli, Collinsella* sp. CAG:289, *Faecalicatena contorta*, and *Clostridium* sp. D5 (Supplementary Fig. 9).

### Analysis of IAD conserved residues

**Fig. 5:**
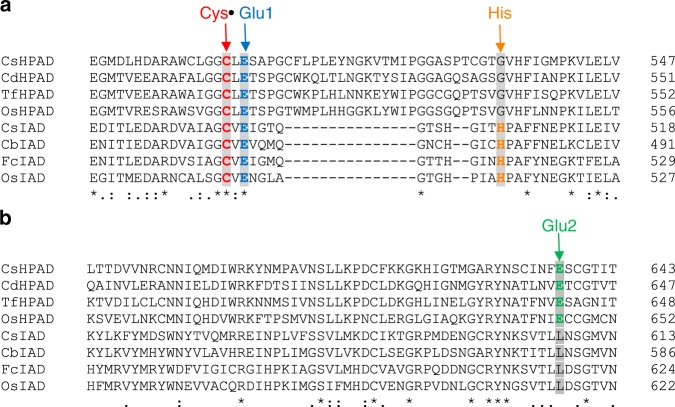
Multiple-sequence alignments of HPADs and IADs, highlighting key residues. **a** Thiyl radical loop region containing conserved Cys• and Glu1 residues, highlighted in red and blue, respectively. A His residue possibly involved in the catalytic mechanism of IAD is coloured orange (Cd *Clostridium difficile*; Tf *Tannerella forsythia*; Cb *Clostridium botulinum*; Fc *Faecalicatena contorta*). **b** Region containing Glu2, highlighted in green. Glu2 is conserved in HPADs but not in IADs

The mechanism of *p*-hydroxyphenylacetate decarboxylation by HPAD has been extensively investigated, both experimentally^24^ and computationally^25^. To investigate the possible mechanism of indoleacetate decarboxylation, sequence alignments between selected HPADs and putative IADs were constructed using Clustal Omega^36^ (Fig. 5a, b), and key residues involved in catalysis were examined. Both HPAD and IAD contain the G• and cysteine thiyl radical (Cys•) residues conserved in all GREs. In addition, the mechanism of HPAD is thought to involve a Glu that coordinates the Cys• (Glu1), and a Glu that coordinates the substrate *p*-hydroxy group (Glu2)^25^. IAD contains Glu1, but not the substrate-coordinating Glu2, consistent with the different substrates of these two enzymes.

The crystal structure of CsHPAD in complex with its substrate *p*-hydroxyphenylacetate showed a direct interaction between the substrate carboxylate group and the thiyl radical residue^24^. To investigate whether IAD might bind its substrate in a similar orientation, a homology model was constructed for OsIAD using CsHPAD as a template (32% sequence identity between the two proteins), followed by docking of the indoleacetate substrate. The model suggested that indoleacetate is bound in a similar conformation as hydroxyphenylacetate in CsHPAD: the acetate group has almost the same conformation, and the indole ring is more or less in the same plane as the phenol ring (Supplementary Fig. 10). The OsIAD residue His514, which is conserved in IAD but not in HPAD (Fig. 5a), could form a hydrogen bond with the indole N-H (Supplementary Fig. 10). However, given the low homology between the modelled protein and the template, further structural studies are required and are currently underway.

## Discussion

The identification of IAD adds to the diversity of enzyme-catalysed radical-mediated decarboxylation reactions. Decarboxylation of arylacetates is chemically difficult, as direct elimination of CO_2_ leaves an unstable carbanion. For HPAD, decarboxylation is promoted by 1-electron oxidation of *p*-hydroxyphenylacetate through a proton-coupled electron transfer (PCET) mechanism that is unique among GREs^24^. In the substrate activation step, the transfer of an electron from the substrate to the Cys•–Glu1 dyad is accompanied by the concerted transfer of the phenolic-OH proton from the substrate to Glu2^24^, generating a phenoxy-acetate anion radical intermediate that subsequently undergoes decarboxylation. An analogous PCET mechanism for IAD would require the transfer of the indolic-NH proton to a suitably positioned base, generating an indoleacetate anion radical intermediate. Our homology model suggests His514 as a candidate base to fulfil such a role (Supplementary Fig. 10). Further structural and biochemical studies, which are clearly needed to investigate the catalytic mechanism, are currently underway.

The fact that IAD tends to occur in bacteria with HPAD (Supplementary Fig. 9) suggests that the two decarboxylases may share a common physiological function. A function that has been suggested for GRE decarboxylases is alkalinization of the cytoplasm for pH regulation in acidic environments, or generation of a proton motive force^6^. This proposal is consistent with the observation that two prolific cresol/skatole-producing organisms *C. scatologenes* and *C. drakei* were isolated from acidic sediments^8^. The production of the bacteriostatic *p*-cresol by *C. difficile* has also been proposed to confer an advantage over its competitors, due to its high level of tolerance to the compound^7^. Skatole has been reported to have broad bacteriostatic effects^10^ and might serve a similar function in skatole-producing bacteria, though more investigations are clearly needed.

The discovery of IAD provides a marker for the identification of skatole-producing bacteria. This is especially significant because there is no systematic method for enrichment culture of skatole-producing bacteria and, despite the conspicuous presence of skatole in human/animal-associated environments, *Os* is the only skatole-producing bacterium isolated from an animal source to date. Our analysis (Supplementary Fig. 9) revealed the presence of IAD sequences in a further two bacteria of human origin: *Olsenella uli* DSM 7084 from human gingival crevice^37^, and *Faecalicatena contorta* from gangrenous appendicitis^38^, highlighting its relevance to human health. In particular, its presence in the oral microbiome implicates its contribution to halitosis^39^.

## Methods

### Materials

Luria Bertani (LB) media was purchased from Oxoid Limited (Hampshire, UK). Acetonitrile was purchased from Concord Technology (Minnesota, USA). Formic acid was purchased from Merck (New Jersey, USA). Skatole, *S*-(5′-Adenosyl)-l-methionine iodide, and 5′-deoxyadenosine were purchased from Sigma Aldrich (Saint Louis, USA). Trifluoroacetic acid and 2,3-dimethylindole were purchased from J&K (Beijing, China). Talon resin was purchased from Clonetech laboratories Inc. (California, USA). All protein purification chromatographic experiments were performed on an ÄKTA pure or ÄKTA prime plus FPLC machines equipped with appropriate columns (GE Healthcare, USA). Protein concentrations were calculated from the absorption at 280 nm measured using an Eppendorf BioPhotometer^®^ D30. Anaerobic experiments were conducted in a Lab2000 glovebox (Etelux) under an atmosphere of N_2_ with less than 10 ppm O_2_.

### Cloning and expression and purification

Codon-optimized gene fragments of IAD and IADAE were synthesized by General Biosystems, Inc. and were inserted into vectors pET-28a-HT and pET-28a-HMT, respectively. The former contained a His_6_-tag and a Tobacco Etch Virus (TEV) protease cleavage site, followed by the construct of interest while the latter contained, in tandem, a His_6_-tag, maltose-binding protein (MBP) and a TEV protease cleavage site, followed by the construct of interest^40^ both at the *Ssp*I restriction sites, using the Gibson Assembly^®^ Cloning protocol (New England Biolabs, Ipwich, MA, USA). For IAD expression, *E. coli* BL21 (DE3) cells (New England Biolabs, Ipwich, MA, USA) were transformed with the plasmid HT-IAD, and grown in LB supplemented with 50 μg/mL kanamycin. For IADAE expression, *E. coli* BL21 (DE3) cells were co-transformed with plasmids HMT-IADAE and pGro7 (TaKaRa, for the co-expression of groES-groEL chaperone), and grown in LB medium supplemented with 50 μg/mL kanamycin, 25 μg/mL chloramphenicol and 0.5 mg/mL l-arabinose. Both cultures (typically 1 L in a 2.6 L flask) were grown at 37 °C while being shaken at 220 rpm. When OD_600_ reached ∼0.8, the temperature was decreased to 16 °C and isopropyl *β*-d-1-thiogalactopyranoside was added to a final concentration of 0.3 mM to induce the production of the proteins. Cells were harvested by centrifugation (4000 × *g* for 10 min at 4 °C) after 16 h.

Cells (~1 g wet weight) were resuspended in 5 mL of lysis buffer [50 mM Tris-HCl, pH 8.0, 1 mM phenylmethanesulfonyl fluoride, 0.2 mg/mL lysozyme, 0.03% Triton X-100, and 1 μL of DNase I (Roche, Germany)]. The cell suspension was frozen in a −80 °C freezer, and then thawed and incubated at room temperature for 50 min to allow cell lysis. 15 mL of buffer A [20 mM Tris-HCl, pH 7.5, and 5 mM β-mercaptoethanol (BME)] containing 1.3% streptomycin sulfate was added, and the precipitated DNA was removed by centrifugation (20,000 × *g* for 5 min at 4 °C). The supernatant was loaded onto a 10 mL TALON column, pre-equilibrated with buffer B [20 mM Tris-HCl, pH 7.5, 5 mM BME, and 0.2 M KCl]. The column was washed with 10 column volumes (CV) of buffer B and then the protein was eluted with 5 CV of buffer B containing 150 mM imidazole. The eluted protein was precipitated with solid (NH_4_)_2_SO_4_ to 70% saturation and isolated by centrifugation (20,000 × *g* for 10 min at 4 °C). The pellet was dissolved in 0.5 mL of buffer B and desalted using a G25 column (GE, USA, thermostat jacket tube XK16/20, packed 15 cm × 2 cm^2^, 30 mL), pre-equilibrated with buffer B. The eluted proteins were concentrated to ~400 μL by ultrafiltration (Sartorius VIVASPIN TURBO 15 (30,000MWCO, Germany)), frozen in aliquots with liquid nitrogen, and stored at −80 °C until further use. The purified IAD (*ε*_280_ = 155,160 M^−1^ cm^−^^1^) and MBP-IADAE (*ε*_280_ = 89,730 M^−1^ cm^−1^) were examined on a 10% SDS-PAGE gel (Supplementary Fig. 1).

### Reconstitution and characterization of IADAE [Fe-S] clusters

A solution of MBP-IADAE (50 μM) was degassed on a Schlenk line and brought into the glovebox. The reconstitution buffer contained 10 mM dithiotheritol (DTT) and 100 mM Tris-HCl, pH 7.5. A solution of ferrous ammonium sulfate (12 eq.) was added followed by a solution of sodium sulfide (12 eq.). The mixture was incubated overnight at 4 °C in a cooling-heating block (Dry Bath H_2_O^3^-100C; Coyote Bioscience, Beijing, China). A solution of EDTA (12 eq.) was then added, and excess of iron and sulfide removed by repeated concentration with a centrifugal filter unit (1.5 mL Ym-30 Amicon; Millipore), and dilution with buffer containing 20 mM Tris-HCl, pH 7.5 and 0.1 M KCl.

The iron contents of as-isolated and reconstituted MBP-IADAE were determined using ferrozine (3-(2-pyridyl)-5,6-diphenyl-1,2,4-triazine-p,p′-disulfonic acid monosodium salt), according to a previously published procedure^41^. The standard curve was established in the range 0–600 μM with Iron Standard for AAS (TraceCERT^®^, Fluka catalogue #16596). For the assay, 50 μL of protein sample (50 μM) was mixed with 100 μL of 2 M HCl, denatured in a boiling water bath for 10 min, and centrifuged for 5 min to remove the precipitated protein. After cooling to room temperature (RT), saturated ammonium acetate (150 μL), freshly prepared 10 mM sodium ascorbate (150 μL), and 10 mM ferrozine (200 μL) were added. Two hundred microlitres of this mixture was transferred to a 96-well plate and A_562_ was monitored with a Tecan M200 plate reader (Switzerland). The readings were tabulated and compared with the standard curve for iron quantitation (Supplementary Fig. 3).

The sulfide contents of as-isolated and reconstituted MBP-IADAE were determined by measuring the absorbance of methylene blue formed upon reaction with *N*,*N*-dimethyl-*p*-phenylenediamine dihydrochloride (DPD)^42,43^.

To obtain the UV–vis absorption spectra, a solution of reconstituted MBP-IADAE was diluted to 10 μM with buffer containing 20 mM Tris/HCl, pH 7.5, 100 mM KCl, and transferred into a septum-sealed anaerobic cuvette (Starna Cells, Quartz Septum Cell) before being taken out of the glovebox. Absorption spectra were acquired in the 200–800 nm range using a Hitachi U3900 spectrometer (Japan). To obtain the spectrum of reduced MBP-IADAE, solution of Ti(III) citrate (10 eq.) was injected using a Hamilton air-tight syringe and incubated for 5 min prior to absorbance measurement. The UV–Vis absorption spectra exhibited features characteristic of [4Fe-4S]^2+^ clusters, which disappeared upon reduction with titanium. Correcting for the 80% purity of MBP-IADAE, the extinction coefficient of the reconstituted MBP-IADAE [4Fe-4S] clusters was estimated to be 24 mM^−1^ cm^−1^ (Supplementary Fig. 4). Given the approximate *ε*_410nm_ of 15 mM^−1^ cm^−1^ per cluster^44^, we estimate that approximately 1.6 [4Fe-4S] clusters per monomer were reconstituted, consistent with the measured Fe and S contents.

### HPLC analysis of SAM cleavage by IADAE

A reaction mixture (400 μL total volume) containing 20 mM Tris-HCl, pH 7.5, 100 mM KCl, 400 μM Ti(III) citrate, and 40 μM reconstituted MBP-IADAE was incubated for 5 min at RT in the glovebox to allow reduction of IADAE. SAM (1 mM final concentration) was added to initiate the cleavage reaction. A control assay omitting Ti(III) citrate was also performed. The reaction was incubated at RT in the glovebox overnight and quenched with formic acid (5% (v/v) final concentration). The reaction mixture was then incubated in a boiling water bath for 45 s to completely denature the protein. The precipitated protein was removed by centrifugation at 14000 × *g* for 10 min. The supernatant was filtered through a 0.22 μm polyethersulfone (PES) membrane (BioRobust). A 20 μL portion of the supernatant was analysed by HPLC (Dionex Ultimate 3000) on a C18 column (Advantage ECHELON C18 4 μm 150 × 2.1 mm P/N: ADV8010, manufactured by ANALYTICAL). The solvent system consisted of 0.1% (v/v) trifluoroacetic acid in water (A) and 0.1% (v/v) trifluoroacetic acid in acetonitrile (B), and the sample was eluted with a linear gradient of 0–16% B over 30 min, with a flow rate of 1 mL/min. The products were detected by UV absorption at 257 nm, and 5′-dA was identified by co-elution with an authentic standard (Sigma) (Supplementary Fig. 5).

### EPR detection of IAD glycyl radical formation

Continuous wave X-band EPR spectroscopy was used to characterize the IAD glycyl radical. A 250 μL reaction mixture containing 20 mM Tris-HCl, pH 7.5, 0.1 M KCl, 40 μM IAD, 80 μM reconstituted MBP-IADAE, 1 mM SAM, and 200 μM Ti(III) citrate was incubated at RT for 10 min inside the glovebox. A control sample omitting Ti(III) citrate was also prepared. A 200 μL portion of each sample was mixed with 50 μL of 50% glycerol, loaded into EPR tubes with 4 mm o.d. and 8″ length (Wilmad Lab-Glass, 734-LPV-7), sealed with a rubber stopper, and frozen in liquid nitrogen prior to EPR analysis.

Perpendicular mode X-band EPR spectra were recorded using a Bruker E500 EPR spectrometer. Data acquisition was performed with Xepr software (Bruker). The experimental spectra for the glycyl radical were modelled with Bruker Xepr spin fit to obtain *g* values, hyperfine coupling constants, and line widths^45^. Double integration of the simulated spectra was used to measure spin concentration based on the equation:

1 $${\mathrm {DI}} = \frac{{{c}}}{{{{f}}\left( {B_1,B_{\mathrm m}} \right)}} \times (G_{\mathrm {R}} \times C_{\mathrm{t}} \times {{n}})[\sqrt P \times B_{\mathrm m} \times Q \times n_{\mathrm B} \times S \times \left( {S + 1} \right)] \times n_{\mathrm S},$$

where DI = double integration; *c* = point sample sensitivity calibration factor; *f*(*B*_1_, *B*_m_) = resonator volume sensitivity distribution; *G*_R_ =  receiver gain; *C*_t_  =  conversion time/s; *P* = microwave power (W); *B*_m_ =  modulation amplitude (G); *n*_B_  =  Boltzmann factor for temperature dependence; *S* = total electron spin; *n* = number of scans; *Q* = quality factor of resonator; and *n*_s_  =  number of spins.

The EPR spectra represent an average of 30 scans and were recorded under the following conditions: temperature, 90 K; centre field, 3370 Gauss; range, 200 Gauss; microwave power, 10 μW; microwave frequency, 9.44 MHz; modulation amplitude, 0.5 mT; modulation frequency, 100 kHz; time constant, 20.48 ms; conversion time, 30 ms; scan time, 92.16 s; receiver gain, 43 dB. Based on our spin quantitation, 0.29 radicals per IAD dimer were formed (Fig. 4).

### GC-MS detection of skatole formation by IAD

The skatole product was quantified by extraction with ethyl acetate, followed by GC-MS analysis. To produce a standard curve, aqueous solutions of skatole (1–5 mM, 300 μL) were extracted with an equal volume of ethyl acetate containing 2,3-dimethylindole (2.5 mM) as an internal standard. The organic phase was then subjected to GC-MS analysis (Supplementary Fig. 6).

GC-MS analysis was performed on a Shimadzu QP2010 GC-MS system operating in ion scan mode (scan range: *m*/*z* 50–700). Samples were chromatographed on a Rxi-1ms (30 m × 0.25 mm ID × 0.25 μm df) column. The injector was operated in split ratio 90:1 mode with the injector temperature maintained at 250 °C. Helium was used as the carrier gas with a flow rate of 1.48 mL/min. The oven programme for the Rxi-1ms column was: ramp of 15 °C/min from 80 to 250 °C, held 3 min. In total ion count (TIC) mode, two peaks were observed with retention times of 5.85 and 6.75 min, corresponding to skatole and the 2,3-dimethylindole standard, respectively (Supplementary Fig. 6). The integral of the skatole TIC peak was normalized by that of 2,3-dimethylindole standard, and the standard curve was obtained by plotting the normalized integral against the corresponding skatole concentration.

For analysis of the IAD reaction, a reaction mixture (300 μL total volume) containing 20 mM Tris-HCl, pH 7.5, 0.1 M KCl, 1 μM IAD, 2 μM IADAE, 0.2 mM Ti(III) citrate, 1 mM SAM, and 1.5 mM (for complete turnover assay Figs. 4b) or 5 mM (Fig. 4c, d) indole-3-acetic acid potassium salt (IAAK) was incubated at 30 °C for 30 min inside the glovebox, followed by quantitation of the skatole product by extraction with ethyl acetate containing 2,3-dimethylindole (2.5 mM) as an internal standard, and GC-MS analysis as described above. Negative controls omitting either SAM or IAAK were also prepared. Skatole produced in the full reaction mixture was identified by co-elution with the authentic standard and confirmed by EI-MS (*m*/*z*), demonstrating that IAD catalysed the decarboxylation of IAAK to form skatole (Fig. 4c, d). The skatole peak was absent in both negative controls. Controls were carried out in parallel, in which IAAK was replaced with *p*-hydroxyphenylacetic acid or phenylacetic acid potassium salts (HPAAK or PAAK, respectively) resulting in no formation of the analogous decarboxylation products *p*-cresol (<0.001 mM) or toluene (<0.03 mM), as monitored by GC-MS.

To determine the *k*_cat_ and *K*_M_ for IAD, 300 μL of reaction mixtures containing 20 mM Tris-HCl, pH 7.5, 0.1 M KCl, 50 nM IAD, 100 nM IADAE, 0.2 mM Ti(III) citrate, 1 mM SAM and varying concentrations of IAAK (0.5, 1.0, 1.5, 2.0, 3.0, 5.0, and 10 mM) were incubated inside the glovebox for 10 min at 30 °C (Supplementary Fig. 7). For time-dependent assays, reaction mixtures containing 1 μM IAD and 10 mM IAAK were incubated at 30 °C inside the glovebox for different time periods (0–60 min). The samples were processed for GC-MS analysis as described above.

### Phylogenetic tree construction

The maximum likelihood phylogenetic tree of *Cs*, *Os*, and previously validated GREs was reconstructed using PHYML^46^ with the LG substitution model^47^. The known enzymes and their UniProt accession IDs are: *Escherichia coli* PFL (P09373), *Lactococcus lactis* PFL (O32797), *E. coli* TdcE (P42632), *Desulfovibrio alaskensis* CutC (Q30W70), *Klebsiella pneumoniae* CutC (A0A0M3KL44), *Roseburia inulinivorans* PDH (Q1A666), *Clostridium butyricum* GDH (Q8GEZ8), *Clostridium difficile* HypD (A0A031WDE4), *Thauera aromatica* BssA (O87943), sulfate-reducing bacterium AK-01 AssA (B8FEM4), *Clostridium difficile* HPAD (Q18CP5), *Clostridium scatologenes* HPAD (A0A0E3JS98), *Tannerella forsythia* HPAD (A0A1D3UC78). The sequence for *Acidobacterium* PhdB was taken from ref. ^6^. The unequal rate of variation among amino acid sites was modelled with a γ distribution with a shape parameter^48^ of 1.3. The level of confidence for the branches was determined based on 100 bootstrap replicates^49^. The resulting consensus tree was rendered using the web-based programme iTOL^50^.

### Homology model construction

The homology model of OsIAD was made by using CsHPAD (PDB: 2YAJ [10.2210/pdb2YAJ/pdb]) as the template. The sequence alignment between OsIAD and CsHPAD sequences was made by MAFFT version 7 (32% sequence identity between the two proteins). The indoleacetate molecule was docked into the active site of the OsIAD model by Induced-Fit Docking workflow in Schrodinger Suite 2017-3. For the nine motifs that form the active site, all of them can have gapless alignment except for L2 and S6 (Supplementary Fig. 10a). Residue Ala396 in L2, His514 in S6, and Leu616 in S7 are only conserved in IAD but not HPAD, their orientations reflect that our model is roughly correct. His514 forms a hydrogen bond with the indole N-H; and the tiny Ala396 replaces Leu400 in CsHPAD, since it faces the bulkier indole ring. Leu616 replaces Glu637 in CsHPAD, forming hydrophobic interactions with the indole ring. Put simply, the model we built is reasonable. The position of Cys500, Glu502, and Gly853 are expected to be similar to the corresponding residues in CsHPAD, although confidence in the position of the other substrate-interacting residues is limited by the low homology between the modelled protein and the template, the position of several key residues such as Ala396, His514, and Leu616 can be justified.

## Electronic supplementary material

Supplementary Information

## Data Availability

Data supporting the findings of this manuscript are available from the corresponding authors upon reasonable request.
