# Supplementary material for: Indoleacetate decarboxylase is a glycyl radical enzyme catalysing the formation of malodorant skatole
Source: Nat Commun. 2018 Oct 11;9:4224. doi: 10.1038/s41467-018-06627-x (PMC6181972; doi:10.1038/s41467-018-06627-x)
Supplement: Supplementary file 1 — Supplementary Information [file 41467_2018_6627_MOESM1_ESM.pdf]

Indoleacetate decarboxylase is a glycyl radical enzyme catalysing the formation of malodorant skatole

Authors: Dazhi Liu[1*], Yifeng Wei[2*], Xuyang Liu[3,4], Yan Zhou[1], Li Jiang[1], Jinyu Yin[1], Feifei Wang[1], Yiling Hu[1], Ankanahalli N Nanjaraj Urs[1], Yanhong Liu[5], Ee Lui Ang[2], Suwen Zhao[3,4†], Huimin Zhao[2, 6†], Yan Zhang[1†]

Affiliations:
[1]Tianjin Key Laboratory for Modern Drug Delivery & High-Efficiency, Collaborative Innovation Center of Chemical Science and Engineering, School of Pharmaceutical Science and Technology, Tianjin University, Tianjin 300072, China.
[2]Metabolic Engineering Research Laboratory, Institute of Chemical and Engineering Sciences, Agency for Science, Technology and Research (A*STAR), Singapore, Singapore.
[3]iHuman Institute, ShanghaiTech University, 201210, Shanghai, China.
[4]School of Life Science and Technology, ShanghaiTech University, 201202, Shanghai, China.
[5]Technical Institute of Physics and Chemistry, Chinese Academy of Sciences, Beijing 100190, China.
[6]Department of Chemical and Biomolecular Engineering, University of Illinois at Urbana-Champaign, 600 South Mathews Avenue, Urbana, Illinois, 61801, USA.

[*] These authors contribute to this work equally

[†]To whom correspondence should be addressed:

Phone: (86) 22-87401835. Fax: (86) 22-87401830. E-mail: yan.zhang@tju.edu.cn

Phone: (217) 333-2631. Fax: (217) 333-5052. E-mail: zhao5@illinois.edu

Phone: (86) 21-20685005. E-mail: zhaosw@shanghaitech.edu.cn

Supplementary Information

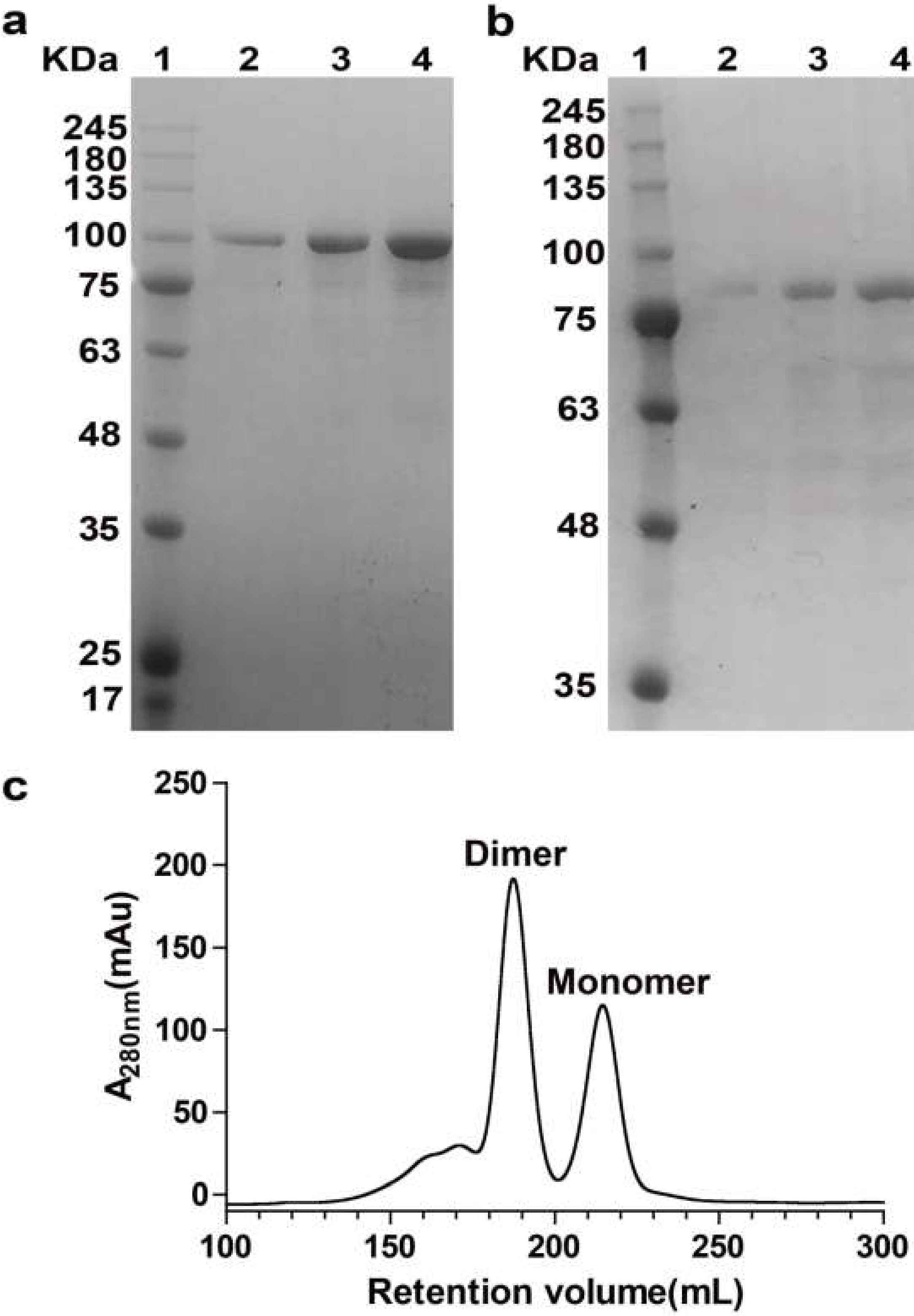

Supplementary Fig. 1 SDS-PAGE and SEC analyses of purified recombinant enzymes. a IAD **b** MPB-IADAE were separated on a 10% SDS gel with: lane 1, protein molecular weight marker; and lane 2-4: 1, 2, 4 μg of IAD or MBP-IADAE. **c** SEC analyses of IAD.

```
OsIADAE     MKNEDGKLKAMIFDIQSFSTHDGPGIRTNVFFKGCTLRCPWCANPESQKGNPQLLYTKMK    60
CsHPADAE    -----MKEKGLIFDIQSFSVHDGPGCRTSVFFIGCPLQCKWCANPESWTKKKHIMVAENV    55
CdHPADAE    -MSSQKQLEGMIFDVQSFSVHDGPGCRTTVFLNGCPLSCKWCANPESWTVRPHMMFSELS    59
TaBssD      ------MKIPLITEIQRFSLQDGPGIRTTIFLKGCPLRCPWCHNPETQDARQEFYFYPDR    54
CbGDHAE     ---MSKEIKGVLFNIQKFSLHDGPGIRTIVFFKGCSMSCLWCSNPESQDIKPQVMFNKNL    57
DaCutD      ----MIERKALIFNIQKYNMYDGPGVRTLVFFKGCPLRCKWCSNPEGQLRQYQVLYKENL    56
CdHypD      -------MNPLVINLQKCSIHDGPGIRSTVFFKGCPLECVWCHNPESQTYTKQVLYNEER    53
EcPflA      -----MSVIGRIHSFESCGTVDGPGIRFITFFQGCLMRCLYCHNRDTWDT----------    45
RiPDHAE     -MKEYLNTSGRIFDIQRYSIHDGPGVRTIVFLKGCALRCRWCCNPESQSFEVETM-----    54
EcNrdG      ---------MNYHQYYPVDIVNGPGTRCTLFVSGCVHECPGCYNKSTWRVNSGQPFTKAM    51
                         .    .  :*** *   *. **   *  * * .

OsIADAE     CI---GCMCCARACPYGAVSAITDPDEIKRVGYVHHDRSKCDKCTTHECLSACFQEALSI    117
CsHPADAE    CKWKNGCRSCINACSHDSIKFSED-------GKLKISWDTCEKCETFDCVNMCPNNALKQ    108
CdHPADAE    CQYENGCTVCHGKCKNGALSFNLD-------NKPVIDWNICKDCESFECVNSCYYNAFKL    112
TaBssD      CV---GCGRCVAVCPAETSRLVRNSDGR---TIVQIDRTNCQRCM--RCVAACLTEARAI    106
CbGDHAE     CT---KCGRCKSQCKSAAIDM-NSE--Y---R---IDKSKCTECT--KCVDNCLSGALVI    103
DaCutD      CV---HCGACVPVCPAGVHTISAST--L---RHGFAEGAQCIGCR--RCEDVCPSSALAV    106
CdHypD      CS---KCEACINICPHKAIYKGETK--I---C---LDQDKCEFCE--TCLDYCVNNAREI    100
EcPflA      -----------------------------------------------------------H    46
RiPDHAE     ----------------------------------------------------TINGKPKV    62
EcNrdG      E-----------------------------------------------------------    52
```

Supplementary Fig. 2 Multiple sequence alignment of N-terminal regions of OsIADAE with previously studied GRE activating enzymes. The three cysteine residues that coordinate the radical SAM [4Fe-4S] cluster[1], present in all the sequences, are coloured blue. For sequences containing an additional ferredoxin-like domain[2], the eight cysteine residues thought to coordinate the two auxiliary [4Fe-4S] clusters in that domain are coloured red. The sequences are for the activating enzymes of: OsIADAE (A0A124EH39) – *Olsenella scatoligenes* IAD, CsHPADAE (UniProt ID: Q38HX2) – *Clostridium scatologenes* HPAD, CdHPADAE (Q84F14) – *Clostridium difficile* HPAD, TaBssD (O87941) – *Thauera aromatic* benzylsuccinate synthase, CbGDHAE (Q8GEZ7) – *Clostridium butyricum* glycerol dehydratase, DaCutD (Q30W71) – *Desulfovibrio alaskensis* choline-trimethylamine lyase, CdHypDAE (A0A069AMK2) – *Clostridium difficile* 4-hydroxyproline dehydratase, EcPflA (P0A9N4) – *Escherichia coli* pyruvate formate lyase, RiPDHAE (Q1A665) – *Roseburia inulinivorans* 1,2-propanediol dehydratase, EcNrdG (P0A9N8) – *Escherichia coli* anaerobic ribonucleotide reductase.

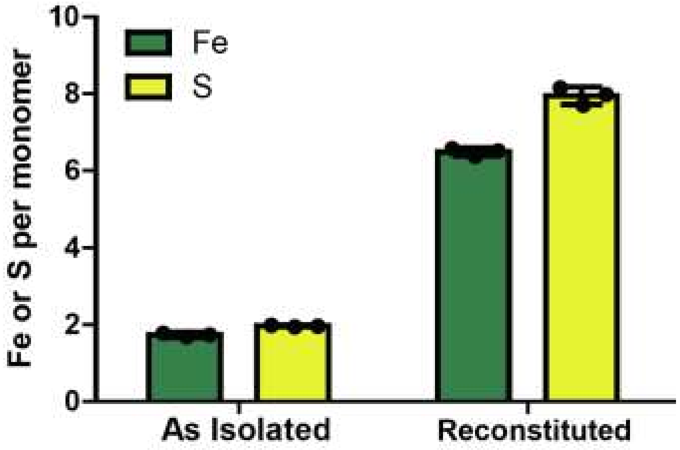

Supplementary Fig. 3 Fe and S contents in MBP-IADAE. The assays were performed in triplicate and presented with standard deviations. Green bars represent Fe contents, yellow bars represent S contents.

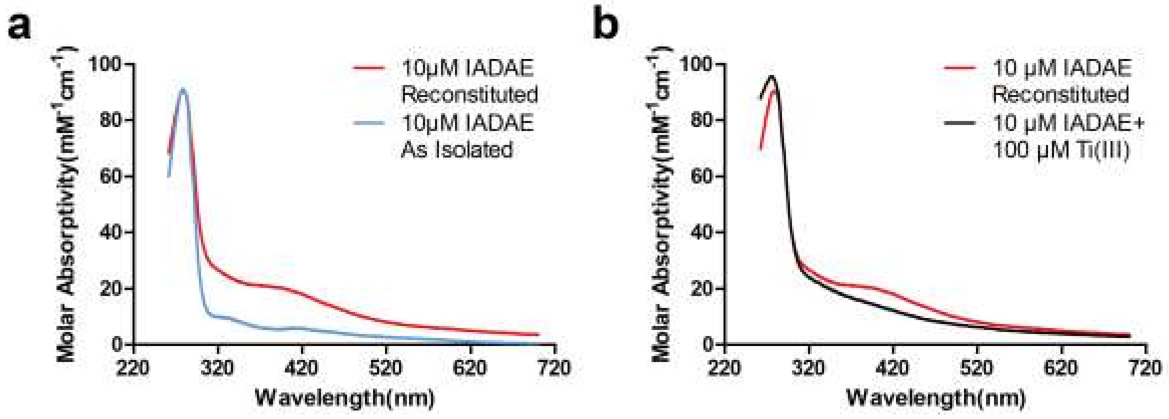

Supplementary Fig. 4 UV-Vis absorption spectra of MBP-IADAE. a Spectra of MBP-IADAE as isolated and reconstituted. **b** Ti(III) was used to reduce the reconstituted $[4Fe-4S]^{2+}$ clusters in MBP-IADAE.

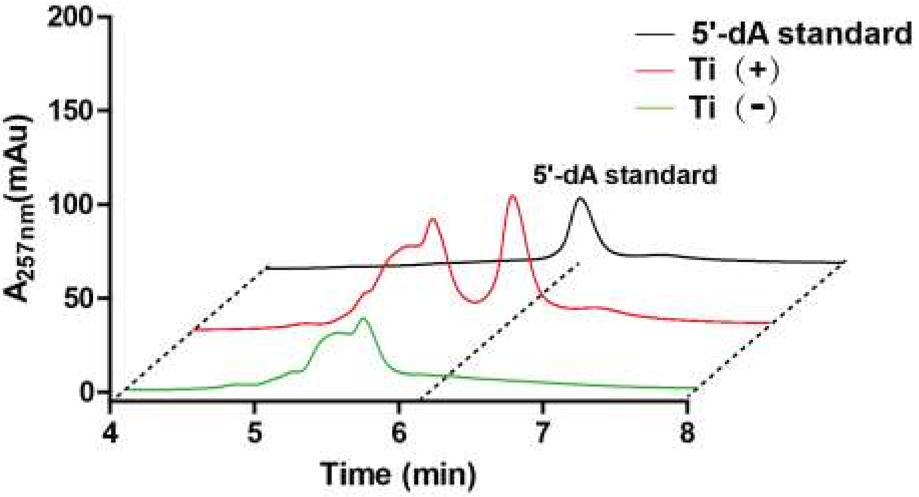

Supplementary Fig. 5 HPLC analysis of the IADAE-catalysed SAM cleavage product, 5'-deoxyadenosine (5'-dA).

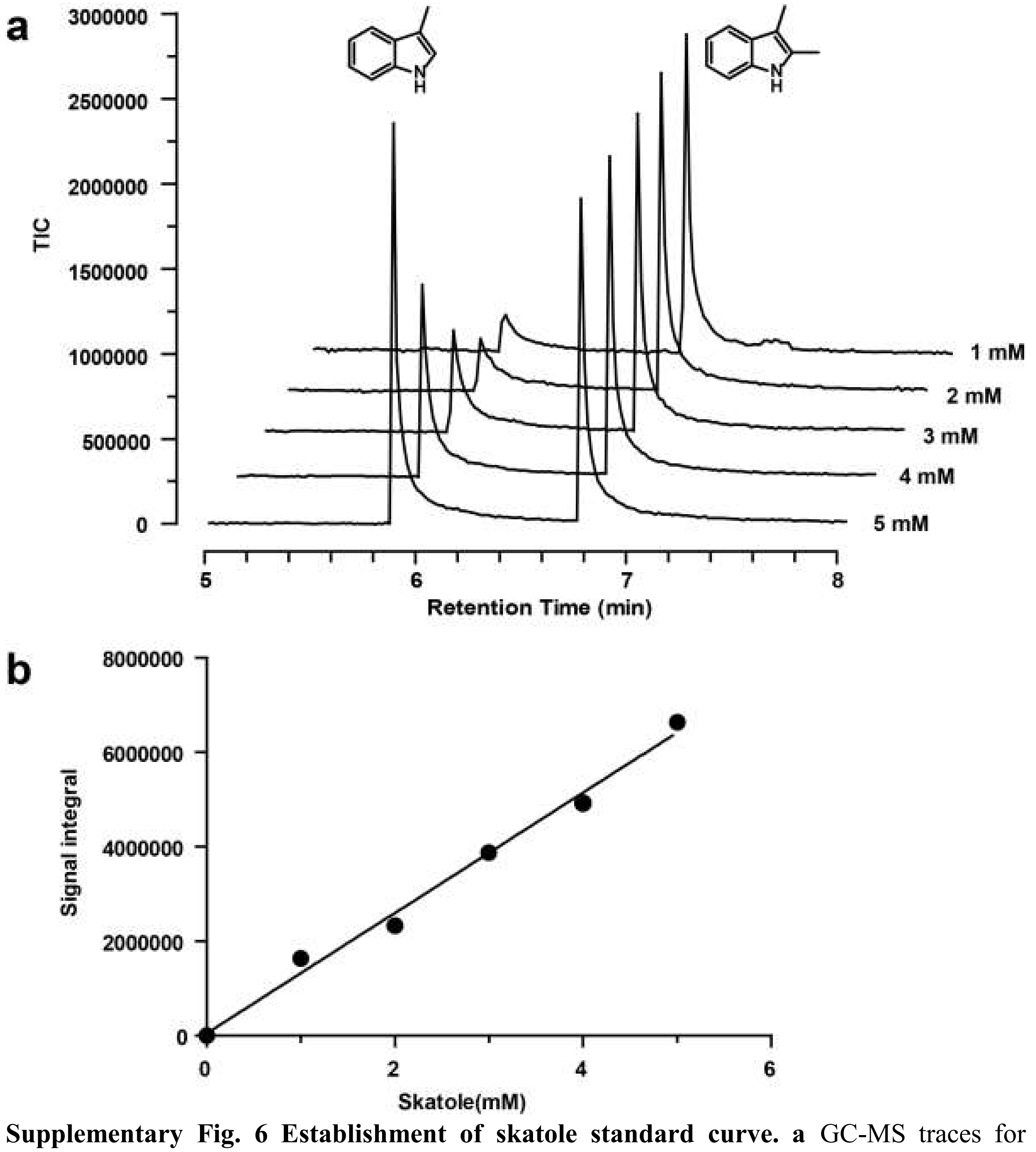

Supplementary Fig. 6 Establishment of skatole standard curve. a GC-MS traces for varying concentrations of skatole and internal standard 2,3-dimethylindole. **b** Standard curve derived from GC-MS traces.

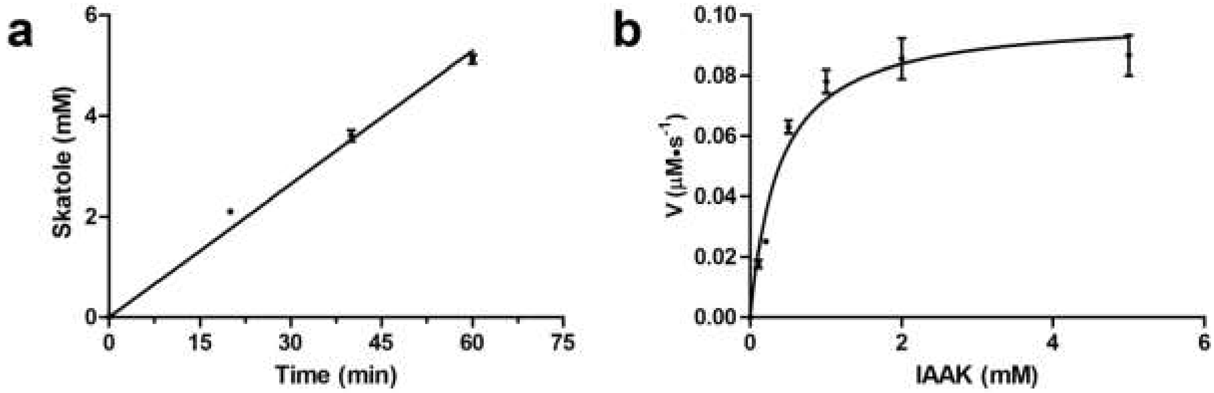

Supplementary Fig. 7 IAD reaction kinetics through monitoring skatole formation by GC-MS. a Time-dependent skatole formation. 1 μM IAD was assayed for activity with 10mM IAAK. **b** Michaelis–Menten kinetics of IAD. 50 nM IAD were assayed for activity with varying IAAK concentrations. The error bars represent the standard deviation of three individual experiments.

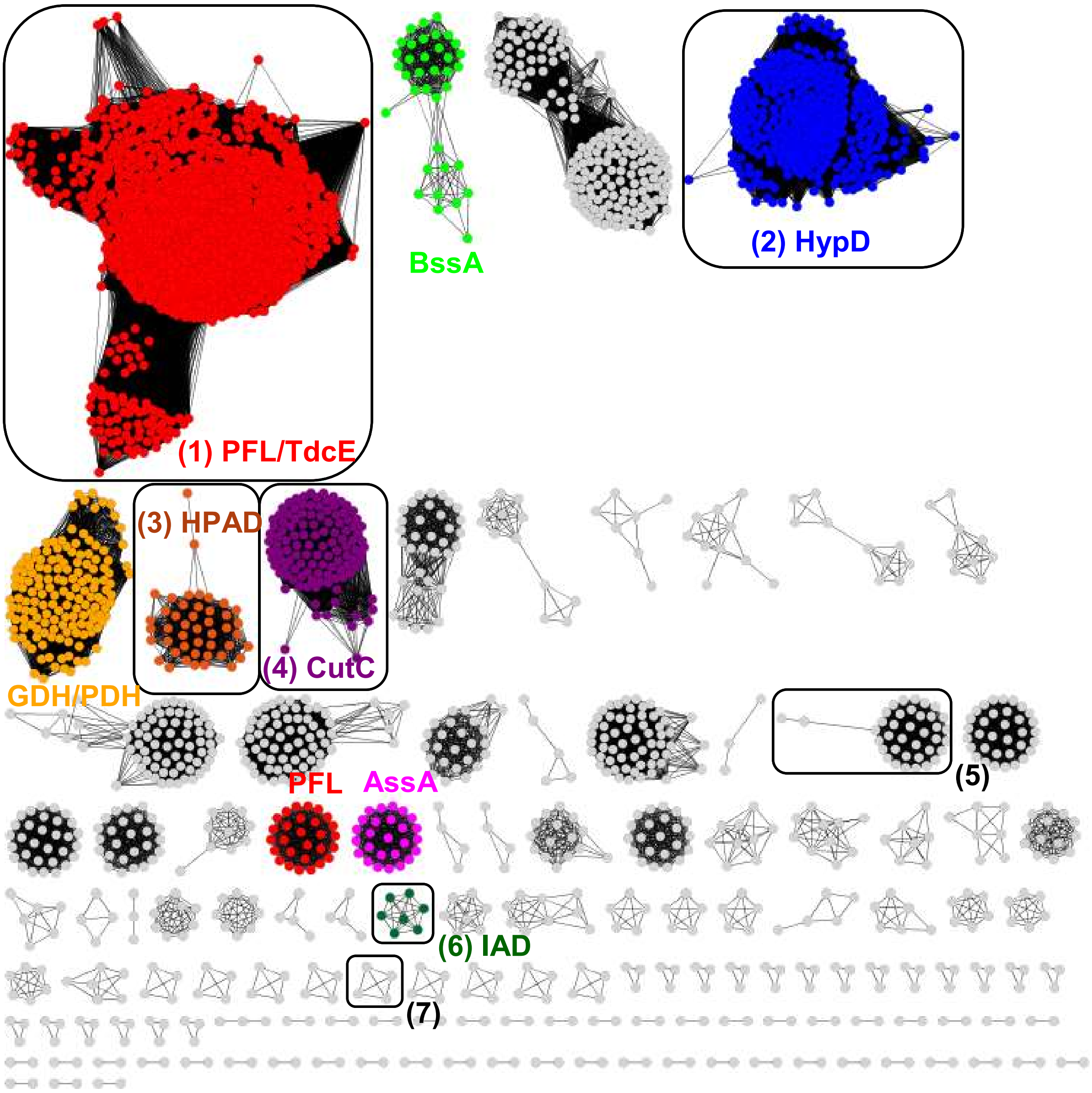

Supplementary Fig. 8 SSN for the GRE superfamily (IPR004184) is displayed at the E-value cut-off of 10^{-260}. The most highly related proteins are grouped together in clusters, putatively sharing the same function. Each node represents a group of proteins sharing more than 90% sequence identity. Clusters containing GREs present in *Clostridium scatologenes* (*Cs*) and *Olsenella scatoligenes* (*Os*) are enclosed in boxes and are displayed in Figure 2. Coloured clusters contain enzymes with known functions, as labelled. The known enzymes and their UniProt accession IDs are: PFL - pyruvate formate lyase (P09373, O32797); TdcE - 2-keto acid formate-lyase (P42632); CutC - choline-trimethylamine lyase (Q30W70, A0A0M3KL44); PDH - propanediol dehydratase (Q1A666); GDH - glycerol dehydratase (Q8GEZ8); HypD - *trans*-4-hydroxy-L-proline dehydratase (A0A031WDE4); BssA - benzylsuccinate synthase (O87943); AssA - alkylsuccinate synthase (B8FEM4); HPAD - *p*-hydroxyphenylacetate decarboxylase (Q18CP5, A0A0E3JS98, A0A1D3UC78); and IAD - indoleacetate decarboxylase (A0A100YXA1), reported in this study.

a

#	Phylum	Organism	IAD UniProt ID	HPAD UniProt ID
1	Actinobacteria	*Olsenella scatoligenes*	A0A100YXA1	A0A100YUQ0
2	Actinobacteria	*Collinsella sp. CAG:289*	R7D0K5	R7D4X2
3	Actinobacteria	*Olsenella uli MSTE5*	X8HS46	X8HWA0
4	Actinobacteria	*Olsenella uli DSM 7084*	E1QXZ2	E1QVI8
5	Firmicutes	*Faecalicatena contorta*	A0A174H1C3	A0A174DZW1, A0A174FUW2
6	Firmicutes	*Clostridium sp. D5*	F0Z0V1, F0YVD2	F0YVT7, F0YZA4
7	Firmicutes	*Clostridium botulinum*	A0A0L7NFY0	A0A0M1LIM4
8	Firmicutes	*Clostridium botulinum C/D str. DC5*	A0A0A0IHA2	-
9	Firmicutes	*Clostridium botulinum C str. Eklund*	B1BA70	-
10	Firmicutes	*Clostridium ragsdalei P11*	A0A1A6ASB6	-
11	Firmicutes	*Clostridium ljungdahlii*	A0A166SJU7	-
12	Firmicutes	*Clostridium scatologenes*	A0A0E3M8P3	A0A0E3JS98

b

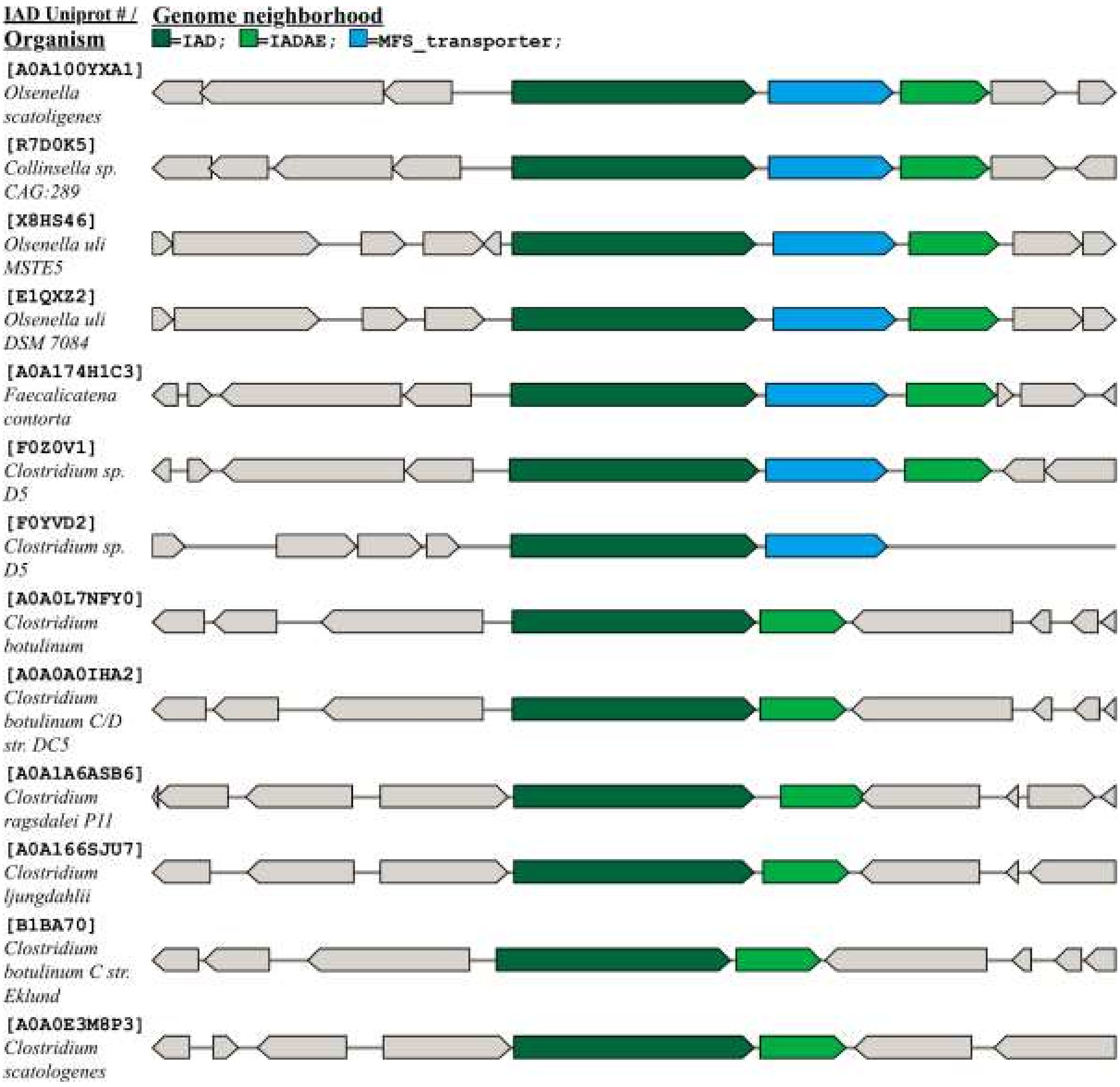

Supplementary Fig. 9 Table of organisms containing candidate IADs and their genome neighbourhoods. a List of the 12 organisms that contain the 13 candidate IADs in the IAD cluster of the SSN (Supplementary Fig. 8, note that *Clostridium* sp. D5 contains two unique IAD sequences). The UniProt accession IDs of the candidate IADs are given. The organisms are bacteria belonging to the phyla Actinobacteria and Firmicutes. Of the 12 IAD-containing organisms, 8 also contain HPAD, and the UniProt accession IDs of the HPADs are given. **b** Genome neighbourhood of candidate IADs (dark green), showing the presence of IADAE (light green). Of the 13 IAD sequences, 7 occur adjacent to an MFS transporter (blue).

a

osIAD numbering	226	341	395 401	500	514	616	730	787	853
	*	*	* *	*	*	*	*	*	*
IAD_A0A100YXA1_os	AGCDGR	LG	YITPSL--WAAMASSNSF	SGCVE	HPIAHPAF	TLL	AALSI	AS	VRVAGF
HPAD_2YAJ_cs	AIPQGR	SG	CFASAG-VSGGVLSGNTF	GGCLE	TCGTGVHF	NFE	CQISV	QN	VRVAGF
HypD_A0A0E3GQ57_cs	EQRGPG	DA	APPKVGVTLKESGTYTDF	SGCVE	EAYILTGY	YIQ	DMLPT	GG	VRVAGY
HypD_A0A125YDI6_cd	EQRAPG	DA	APPKVGITLKESSTYTDF	SGCVE	EAYVLTGY	YIQ	DMLPT	GG	VRVAGY
CutC_5FAU_dd	VNGGGD	TG	WITSEG-ASKFFAGYQPF	MGCVE	YQWTSTGY	GVV	GTLSI	IG	VRVAGY
GDH_1R9D_cb	YNGVGH	HS	KVRDEI-STKHFGGYPMY	IGCVE	EGWHDSAF	GPQ	GLYPS	NG	VRVAGY
PDH_5I2G_ri	YNGVGH	HS	KCRDAA-SAEGFAGYSLF	IGCVE	DGWHDAAF	GPQ	GLYPV	NG	VRVAGY
PhdB_A0A2P6WDZ5_ab	DDMGGM	VH	FVMNPE--LAIWQQSRIA	AGCVQ	TDGTWEAR	GVC	SAYSV	GS	VRVAGF
AssA_B0CMZ9_sb	AYGYSG	EG	GFAPRW-AREGLQGITGT	QACMS	PMRMASAT	WVT	LPENV	RA	VRVAGY
BssA_5BWE_ta	ATGYNS	AS	AGKSRA-YREIFPGSNDL	VLCMA	TRSEGGSA	WHN	TGQAV	KA	VRVSGF
PFL_3PFL_ec	AYGRGR	AA	FLRTPE-YDELFSGDPIW	ACCVS	QMQFFGAR	ACG	SVLTI	DG	IRVSGY
	*	*	* *	*	*	*	*	*	*
ecPFL numbering	176	272	327 333	419	432	525	604	661	734
	S1	S2	L2 S3	TRL	S6	S7	S8	S9	GRL

b

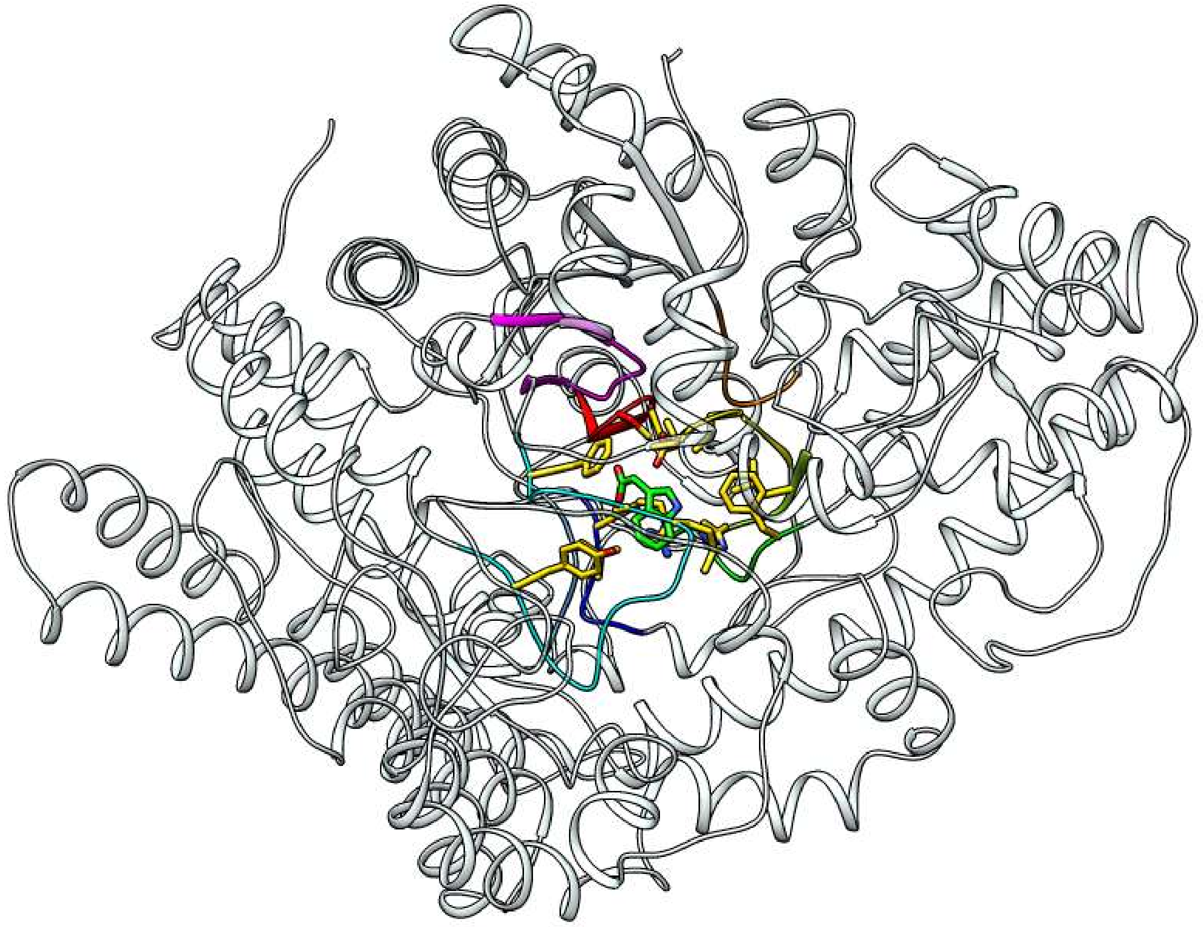

c

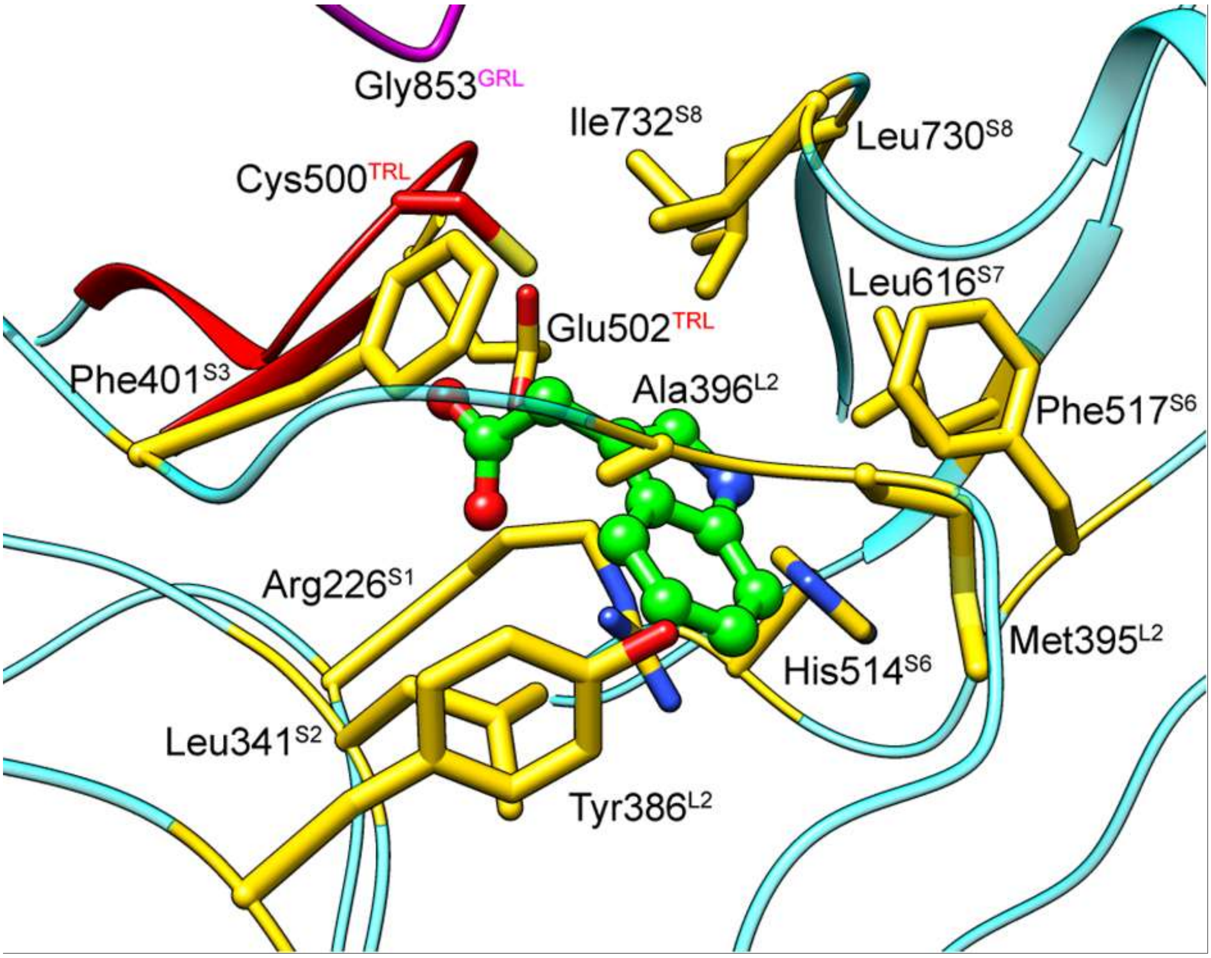

Supplementary Fig. 10 Sequence alignment and homology model of OsIAD. a Multiple sequence alignment of OsIAD with a selection of functionally diverse GREs, including all structurally characterized GREs, used in the construction of the OsIAD homology model. The GREs are labelled by their function, followed by UniProt / PDB accession ID, and organism. Key secondary structural elements forming the active site, including β-strands (S), loops (L), the cysteine thiyl radical loop (TRL), and glycyl radical loop (GRL) are as labelled with coloured bars. The top and bottom numbering of the residues follows that of OsIAD and *E. coli* PFL, respectively. **b** Model of the overall 3-dimensional structure of IAD. **c** Model of the active site of IAD. The indoleacetate substrate is rendered in green. The glycyl radical loop containing the G• cofactor is rendered in purple. The thiyl radical loop including the radical-forming Cys500 residue is rendered in red. Other active-site residues in contact with the substrate are rendered in yellow.

Supplementary References

1. Shisler KA, Broderick JB. Glycyl radical activating enzymes: structure, mechanism, and substrate interactions. *Arch Biochem Biophys* **546**, 64-71 (2014).

2. Selvaraj B, Buckel W, Golding BT, Ullmann GM, Martins BM. Structure and Function of 4-Hydroxyphenylacetate Decarboxylase and Its Cognate Activating Enzyme. *J Mol Microbiol Biotechnol* **26**, 76-91 (2016).
